# Investigation of the impact of sulfur on the properties of CZTS nanomaterials for enhanced supercapacitor performance

**DOI:** 10.1039/d5ra04633e

**Published:** 2025-09-02

**Authors:** Roomul Mushtaq, Mohd Zubair Ansari

**Affiliations:** a Department of Physics, National Institute of Technology Srinagar Hazratbal Srinagar J&K 190006 India mhd.zubair1@gmail.com

## Abstract

Cu_2_ZnSnS_4_ (CZTS) has been synthesised using ethylene glycol as a solvent by the solvothermal method. Preliminary characterisation, like X-ray diffraction, Raman spectroscopy, and FTIR, confirmed the tetragonal structure of CZTS with kesterite phase. In the synthesis, a series of samples with different concentrations of sulfur were produced, accompanied by an in-depth analysis of structural parameters such as crystallite size and strain, utilising both the Scherrer equation and the Williamson–Hall method. Utilisation of field emission scanning electron microscopy in conjunction with energy dispersive X-ray (EDX) has enabled the observation of the evolution of spherical morphology and the decrease in agglomeration as the concentration of sulphur was elevated. The peaks observed in EDX and XPS confirmed the presence of elements and their atomic percentages within the synthesised samples. The Tauc plot was employed to determine the optical band gap; it first increases from 1.60 eV to 1.66 eV and then decreases to 1.56 eV. Additionally, electrochemical investigations were conducted utilising CV, GCD, and EIS experiments. A comparative analysis was conducted for samples with the highest (CZTS8) and lowest (CZTS5) sulphur concentrations. Specific capacitance measurements of 481 F g^−1^ and 316 F g^−1^ were obtained for CZTS8 and CZTS5, respectively. CZTS8 demonstrates an impressive 88% capacitance retention over 2600 cycles, and the extension of the linear portion of the EIS graph indicates that both samples exhibit commendable capacitive behaviour.

## Abstract 2

Cu_2_ZnSnS_4_ (CZTS) has been synthesised using ethylene glycol as a solvent by the solvothermal method.

## Graphical abstract

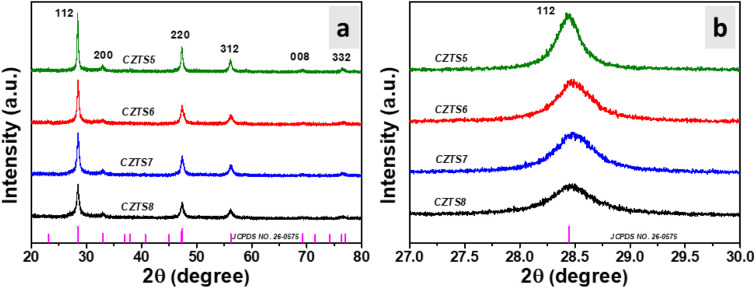

## 1 Introduction

Cu_2_ZnSnS_4_ has been extensively researched for its use in solar cells, in addition to its applications in photocatalysis, photoelectrochemical processes, photodetection, thermoelectric devices, and hydrogen evolution, among others.^1–3^ The outlandish properties that contribute to the diverse applications of CZTS nanomaterials encompass its non-toxicity, cost-effectiveness in production, high absorption coefficient (*α* > 10^4^ cm^−1^) in the visible spectrum, p-type electrical conductivity, and a tunable direct optical bandgap.^4–7^

A quaternary semiconductor compound, Cu_2_ZnSnS_4_ (CZTS), is formed by exchanging indium for zinc and tin in a 1 : 1 ratio, and sulphur for selenium in the original CuInSe_2_ (CIS). The chemical formula of CZTS, more precisely Cu_2_ZnSnS_4_, requires a stoichiometry of 2 : 1 : 1 : 4 for components like copper, zinc, tin, and sulfur. The volatile nature of sulphur presented difficulties when included in most CZTS synthesis procedures, hence its precursor ratio has been taken as 5, 6, 8, or 10 instead of 4 in most of the reported synthesis methods.^8–10^ In certain studies, to uphold the concentration of sulfur, sulphurization was performed to achieve the appropriate ratio of sulfur within the reaction medium.^11,12^ The correct stoichiometry in CZTS leads to phase purity and a distinct morphology. Additionally, an imbalance in sulphur levels can result in the development of secondary and tertiary phases.^13,14^ Although the synthesis of CZTS nanoparticles has already been extensively documented, considerable challenges persist with the materials. The synthesis of this material is intricate due to the multitude of elements involved in the process. The acquisition of a pristine single-phased structure of CZTS presents significant challenges, primarily due to the material's inherently limited compositional range, easy vaporisation of sulfur, and the high potential for secondary phase formation.^15,16^ The precise integration of sulphur in CZTS results in the desired stoichiometric balance, which is essential for obtaining a single-phase structure that optimises the characteristics required for diverse applications, including supercapacitor applications.^5,9,10^

Currently developed materials demonstrate exceptional electrochemical properties essential for addressing the increasing demand for efficient innovative technologies in portable electronics, compact electronic devices, and hybrid electric vehicles.^17^ The boosted power density and extended life cycle of supercapacitors compared to batteries render them a favoured option for primary power sources.^18^ The prospects for energy storage are promising with supercapacitors, which may function as a bridge between capacitors and batteries.^19^ Capacitors, as mechanisms for charge storage, can be categorised into two distinct types: pseudocapacitors and electrochemical double-layer capacitors (EDLCs).^20,21^ Storing charge in pseudocapacitors involves rapid Faradaic redox reactions, which are achieved through the adsorption of ions at the electrode interface in EDLCs, *i.e.*, electric double layer capacitors.^21,22^ Hydroxides, metal oxides, polymers, and nitrogen sulfides represent prevalent materials exhibiting pseudocapacitance. Metal oxides represent the most widely studied class of pseudocapacitive materials.^23,24^ However, the restricted dielectric potential between the electrode and the electrolyte hinders effective electron transport. Metal sulphides, which demonstrate enhanced structural stability relative to oxides, have been utilised to address this issue. This phenomenon can be ascribed to the comparatively lower electronegativity of sulphur about oxygen, which promotes rapid electron transfer.^25^ Quaternary metal chalcogenides arise from combining various metals with at least one chalcogen element, including sulphur, selenium, or tellurium.^26^ Within the realm of quaternary chalcogenides, specifically Cu_2_NiSnS_4_, Cu_2_ZnSnS_4_, and Cu_2_MnSnS_4_, copper zinc tin sulphide (Cu_2_ZnSnS_4_) emerges as an up-and-coming candidate because of its distinguished properties.

In the current work, single-phase synthesis of CZTS is reported. Though the molar ratios of copper, zinc, and tin were held constant, the sulfur molar ratio was optimized to ensure proper stoichiometry. The basic characterisations were carried out to investigate the optimized samples' structural, optical, compositional, and morphological features. The prepared samples functioned as active electrodes to explore the electrochemical characteristics and parameters.

## 2 Experimental

### 2.1 Material synthesis

CZTS nanoparticles were produced through the solvothermal technique, utilising copper sulphide, zinc sulphide, tin chloride, and thiourea as initial precursors in a molar ratio of 2 : 1 : 1 : 5 with ethylene glycol as solvent. The pH of the prepared solution mixture was carefully maintained at approximately 8 through the gradual addition of 2 mmol sodium hydroxide solution. Later, the solution was introduced into a Teflon-lined stainless-steel autoclave, which was then positioned in the oven for 24 hours at a temperature of 200 °C. Following the cooling process, the sample underwent centrifugation at 3500 rpm for 5 minutes, subsequently washed initially with distilled water and then with absolute ethanol. The powder was dried for 12 hours at a temperature of 70 °C before being collected for subsequent characterisation. The nanomaterial produced with this molar ratio has been designated as CZTS5. The steps involved in synthesising the sample are presented in Fig. S1 (SI). Similar steps were followed for the synthesis of other samples in which the molar ratio of sulfur was increased accordingly to get the overall molar ratio, *i.e.*, Cu : Zn : Sn : S as 2 : 1 : 1 : 6, 2 : 1 : 1 : 7, and 2 : 1 : 1 : 8. These samples were respectively named as CZTS6, CZTS7, and CZTS8. These samples' physical and chemical parameters remained unchanged after 15 days of storage at room temperature and humidity before further characterization.

### 2.2 Material characterisation

Cu kα (*λ* = 0.154 nm) X-ray source was used to conduct the X-ray diffraction measurements utilising a Rigaku X-ray diffractometer. The Raman spectroscopy measurements were performed using a Renishaw Raman spectrometer, which employs an argon ion laser (wavelength 514.5 nm). The optical absorption characteristics of CZTS nanoparticles were analysed using a PerkinElmer Lambda 365 UV-Visible spectrophotometer (200–800 nm), and using Tauc plot analysis, the optical band-gap was ascertained. The imaging of the nanoparticles was conducted using a field emission scanning electron microscope (FESEM, Gemini SEM 500) set to an accelerating voltage of 15 kV. The data obtained from energy-dispersive X-ray spectroscopy (EDX) at 20 kV, utilising an EDX connected to a Gemini SEM 500 microscope, facilitated the analysis of the elemental composition of the particles. A Nexsa-Thermofisher spectrometer, utilising monochromatized Al Kα radiation, was employed for the examination of the oxidation states of the materials using X-ray photoelectron spectroscopy (XPS). The XPS peaks underwent deconvolution using the XPSPEAK41 software.

### 2.3 Electrode fabrication

The preparation of the electrode entails several straightforward steps. Initially, a 1 cm^2^ strip of nickel foam was subjected to ultrasonic cleaning for approximately 30 minutes in a dilute hydrochloric acid solution. After thorough washing, the strip was dried in an oven at 65 °C. The specimen was repeatedly weighed and returned to the oven. The repeated weighing confirmed the precise weight of the nickel foam strip, measuring 0.0626 g for the first strip and 0.0652 g for the second strip. To achieve the mass ratio of 85% for active material, 10% for conductive carbon, and 5% for polyvinylidene difluoride (PVDF) as a binder.^27^ We can express the percentage formula as follows:85% = (0.05 g × 100)/*y*

The mass of the active component for slurry preparation was set at 0.05 g, where *y* represents the total mass, including the active material, conductive carbon, and binder mass. According to the equation, the value of *y* is calculated to be 0.0588 g. Employing *y*, we subsequently calculate the mass of conductive carbon and binder to be 0.0058 g and 0.0029 g, respectively. To prepare the slurry, the precise weights of the active material, conductive carbon, and binder were 0.05 g, 0.0058 g, and 0.0029 g, respectively. A mortar and pestle were employed to finely grind the combination of active material, carbon black, and binder into a homogeneous powder. Subsequently, a few drops of *N*-methyl 2-pyrolidone (NMP) solvent were introduced, and the mixture was further ground until an ink-like slurry was achieved. The slurry was applied *via* drop casting onto the nickel foam strip, starting with one side and covering the opposite side. The strip was allowed to dry at room temperature for approximately 5 minutes, after which the slurry was applied again using the drop casting technique. The casting process was reiterated to guarantee that the substance is deposited uniformly into the pores of the nickel foam strip. The electrode underwent an initial drying process at ambient temperature for approximately 30 minutes, followed by an extended drying period in an oven at 65 °C for around 12 hours. The electrode was reweighed upon returning to room temperature, resulting in a mass of 0.0706 g. The mass of the active material mixture (*i.e.*, active material, carbon black, and binder) is determined by subtracting the mass of the bare nickel foam strip, resulting in a value of approximately (0.008 ± 0.0002) g. Before utilising the electrode for experimentation, it underwent a drying process and was subsequently reweighed, revealing no variation in weight. The measured mass of the active substance mixture CZTS5 on Ni-Foam was approximately 8 mg cm^−2^. Analogous methodologies were utilised to fabricate the CZTS8 electrode, resulting in an active mass deposition on Ni-Foam quantified at 8.2 mg cm^−2^. The electrodes that were fabricated underwent electrochemical analysis. The experimental measurements were carried out across a voltage range of 0 to 0.5 V, concerning an Ag/AgCl electrode, employing an aqueous electrolyte solution of 1 M KOH. A ZIVE SP1 potentiostat was employed, set up with a conventional three-electrode cell system, incorporating Platinum as the counter electrode, material deposited on Ni-Foam acting as the working electrode, and Ag/AgCl as the reference electrode.

## 3 Results and discussion

### 3.1 X-ray diffractometer (XRD) analysis

**Fig. 1:**
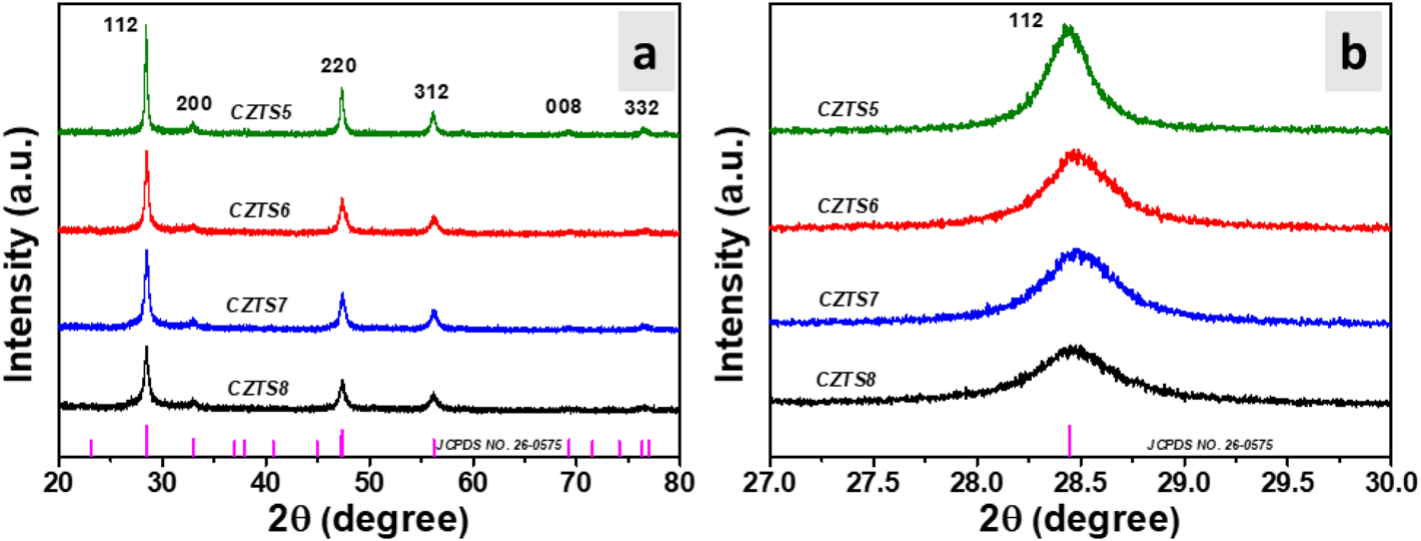
(a) XRD peak pattern of CZTS nanomaterials with different sulfur amounts and (b) (112) central peak of CZTS nanomaterials with varying amounts of sulfur.

**Fig. 2:**
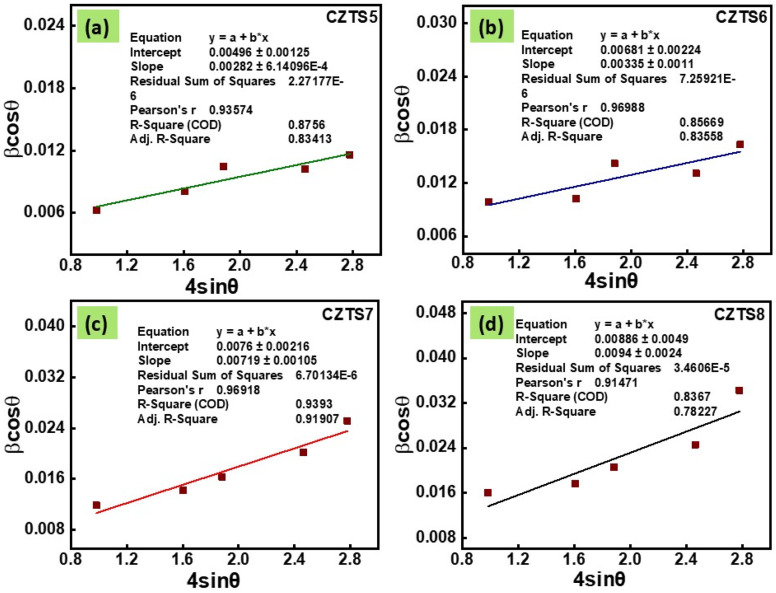
William–Hall plots for CZTS (a) CZTS5, (b) CZTS6, (c) CZTS7, and (d) CZTS8 samples.

The examination of the X-ray diffraction data presented in Fig. 1(a) elucidates critical insights into the crystal structure and phase purity of the material under investigation. The observed spectra display clear peaks corresponding to the crystallographic planes (112), (200), (220), (312), (008), and (332) at diffraction angles of 28.56°, 32.93°, 47.45°, 56.31°, 69.30°, and 76.47°, respectively. The planes in question are indicative of the crystalline tetragonal kesterite phase of CZTS, as documented in JCPDS no. 26-0575.^8,28^ These planes are assigned overlaid vertical lines of the kesterite phase. Alongside the peaks associated with the CZTS phase, there were no indications of additional peaks from supplementary phases like tin sulphide (SnS), copper tin sulphide (Cu_2_SnS_3_), cubic zinc sulphide (ZnS), or orthorhombic Cu_2_SnS_3_. Fig. 1(a) displays the X-ray diffraction peaks for all samples with different optimised sulphur quantities. The existing literature suggests that variations in sulphur concentration can lead to the emergence of secondary phases,^13,14,29^ which was not evident in the XRD study. Fig. 1(b) illustrates that as the sulphur content in the samples increases from a molar ratio of 5 to 8, a slight shift in the most significant peak (112) is observed. Furthermore, it contributes to an increase in peak broadening, as evidenced by the most intense peak (112) depicted in Fig. 1(b). To clarify the influence of sulphur on the crystallinity of synthesised CZTS nanomaterials, parameters such as crystallite size and microstrain have been determined (Fig. 2).

The Scherrer equation presented below was employed to calculate the mean crystallite size for each sample:1
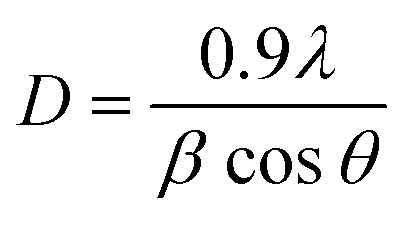


In this context, *β* indicates full width at half maximum (FWHM), the incident X-ray radiation *λ* wavelength is equal to 0.154 nm, and *θ* is the Bragg angle. The crystallite size for CZTS5, CZTS6, CZTS7, and CZTS8 samples calculated by the Scherrer equation is 22.35, 15.75, 14.5, and 11.3 nm, respectively.

A detailed analysis of the diffraction data employing the Williamson–Hall technique was conducted to investigate the fundamental causes of peak broadening and the effect of sulphur concentration on the crystal structure. The intrinsic strain could be a significant factor that affects the structural properties by modifying the size of the crystallites. The crystallite size (*D*) and intrinsic strain (*ε*) for all samples were ascertained through XRD patterns utilising the Williamson–Hall method, based on the subsequent equation:2
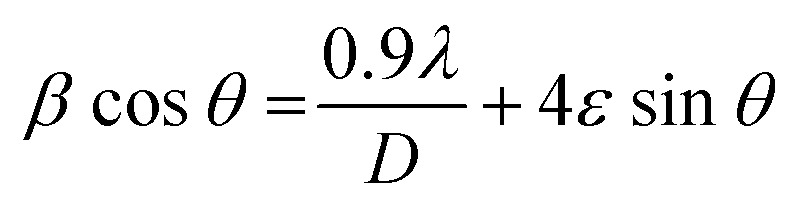


In this context, *β* represents the widening of the diffraction peak at half its peak intensity, *θ* denotes the Bragg angle, and *λ* (0.154 nm) refers to the X-ray wavelength employed. The calculated values of crystallite size and intrinsic strain of all the samples are summarized in Table S1 (SI).

It is essential to observe that the average crystallite size determined through the W–H method exceeds the values acquired *via* the Scherrer formula. The observed increase in crystallite size, as opposed to the Scherrer method, can be attributed to the inclusion of broadening effects resulting from strain (Williamson–Hall method).^30^ Also, this increase in strain is attributed to the rise in defective levels and grain boundaries.^31^ Furthermore, the energy required to maintain a significant crystal increases as strain intensifies. The surplus energy causes the growth of large crystals unfavourably, leading to a rise in grain boundaries and a subsequent decline in crystallinity.

### 3.2 Raman spectroscopy analysis

**Fig. 3:**
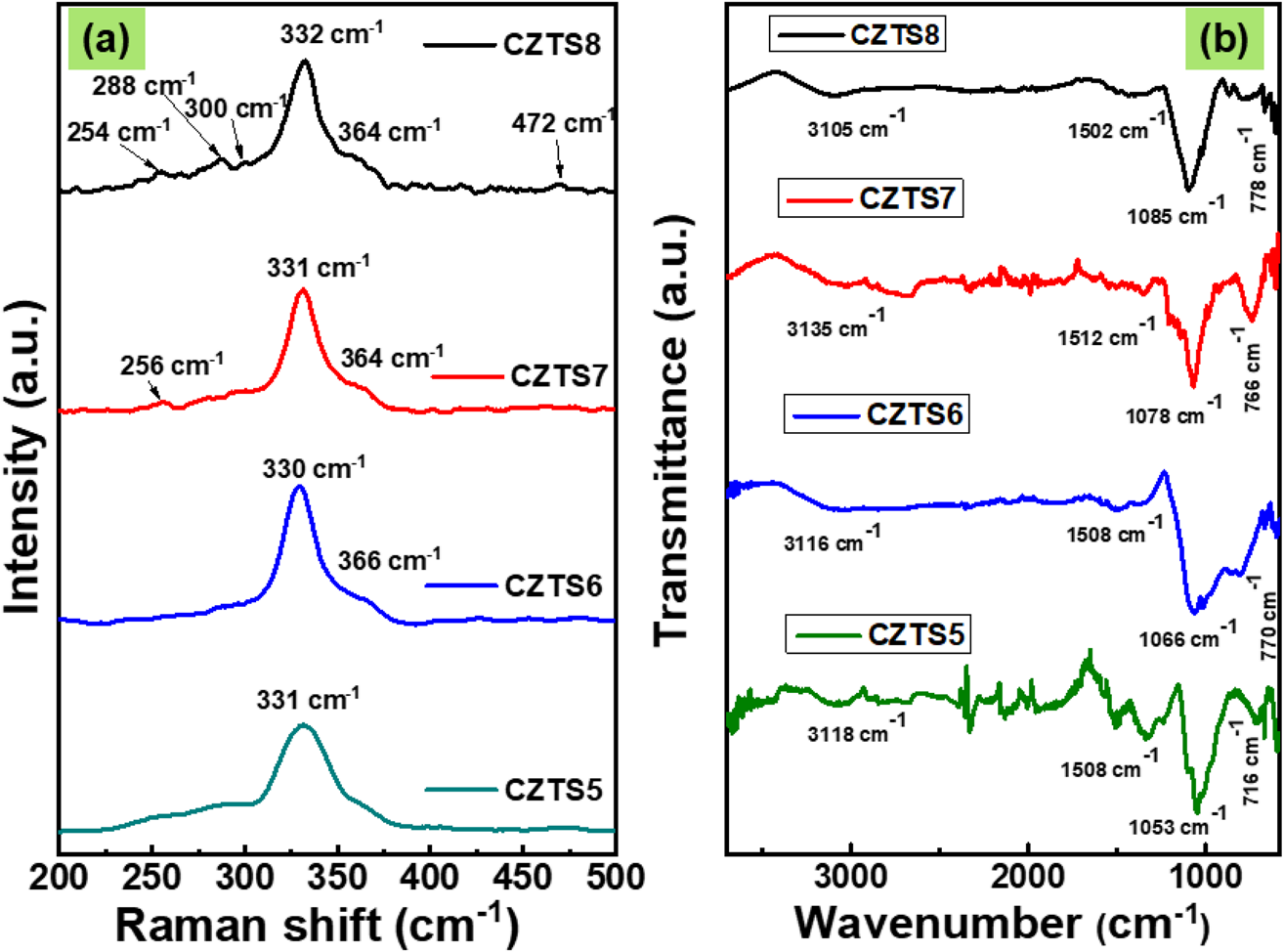
(a) Raman spectra of CZTS5, CZTS6, CZTS7 and CZTS8 nanomaterials (b) FTIR spectra of CZTS5, CZTS6, CZTS7 and CZTS8 samples.

Identifying secondary phases exhibiting analogous crystal structures through X-ray diffraction presents significant challenges, primarily due to their occurrence in negligible quantities. Due to its non-sensitivity to long-range order, Raman spectroscopy has demonstrated significant advantages in identifying secondary phases in CZTS.^32,33^ The Raman spectra recorded at room temperature within the range of 200–500 cm^−1^ using a 514.5 nm laser are presented in Fig. 3(a). According to the literature, the most pronounced peak is observed within the 330–338 cm^−1^.^23^ In the current investigation, all samples exhibit the most pronounced peak within the specified range, with a slight deviation observed for CZTS8; such a shift is related to the disorder in the cation sublattice occurring both in cases of stoichiometry and non-stoichiometry.^33^ Its intense peak is located at 332 cm^−1^; in contrast, the remaining samples display their peaks at 331 cm^−1^, corresponding to the kesterite structure of CZTS. The most pronounced peak is associated with A1 symmetry of CZTS, arising from the vibrational motion of sulphur atoms within the lattice structure. The CZTS7 and CZTS8 samples exhibit a less intense peak at 288 cm^−1^, corresponding to the kesterite phase of CZTS. This peak results from the vibrational modes of sulphur and zinc atoms within the CZTS lattice.^33,34^ A shoulder peak is observed in all the samples at approximately 365 cm^−1^, which is also associated with the tetragonal CZTS phase. In the case of CZTS8, we observed a minor peak at 301 cm^−1^, which corresponded to the Cu_2_SnS_3_ phase. A clear peak is detected in the spectrum at 255 cm^−1^ for the CZTS7 and CZTS8 samples. This peak is related to the longitudinal optical components associated with the polar B and E symmetry modes, with their intensity resulting from the Frohlich electron–phonon interaction.^35^ At 471 cm^−1^, a minor peak of Cu_2−*x*_S phase exists in CZTS8.^36^ Consequently, in all the samples, it is observed that an increase in the *S* concentration leads to the formation of secondary phases, which are less intense when compared to the 332 cm^−1^ peak, as evidenced in the CZTS7 and CZTS8 samples, which were not detected from XRD diffraction. The XRD method can detect the crystalline phases and preferred orientation, but it cannot measure amorphous or sparsely distributed phases. Secondary phases with identical crystal structures are typically challenging to identify using the XRD technique, as is very much possible in the present case. Since Raman spectroscopy is insensitive to long-range order, it has proven helpful in detecting the secondary phases in the CZTS or CZTS-Se absorber materials. One can assess the strain and potential lattice distortions in the crystal by closely examining the peak shift.^33^

### 3.3 FTIR spectroscopy

The influence of different sulphur concentrations on the vibrational modes of copper, zinc, tin, and sulphur was examined by applying FTIR spectrophotometry. The FTIR spectrum for all CZTS samples exhibiting varying sulphur concentrations is presented in Fig. 3(b). The peak detected in the range of 1050–1100 cm^−1^ corresponds to the vibrational mode of NH_2_ in thiourea.^31^ The spectrum displays a wide band within the range of 3105–3135 cm^−1^, attributed to a high concentration of sulphur, with the CZTS8 sample showing the most pronounced peak in this specified range.^37^ This indicates the abundance of sulphur in the CZTS8 sample. An intense peak in the range of 1050–1100 cm^−1^ confirms the formation of CZTS.^38^

### 3.4 Optical analysis

**Fig. 4:**
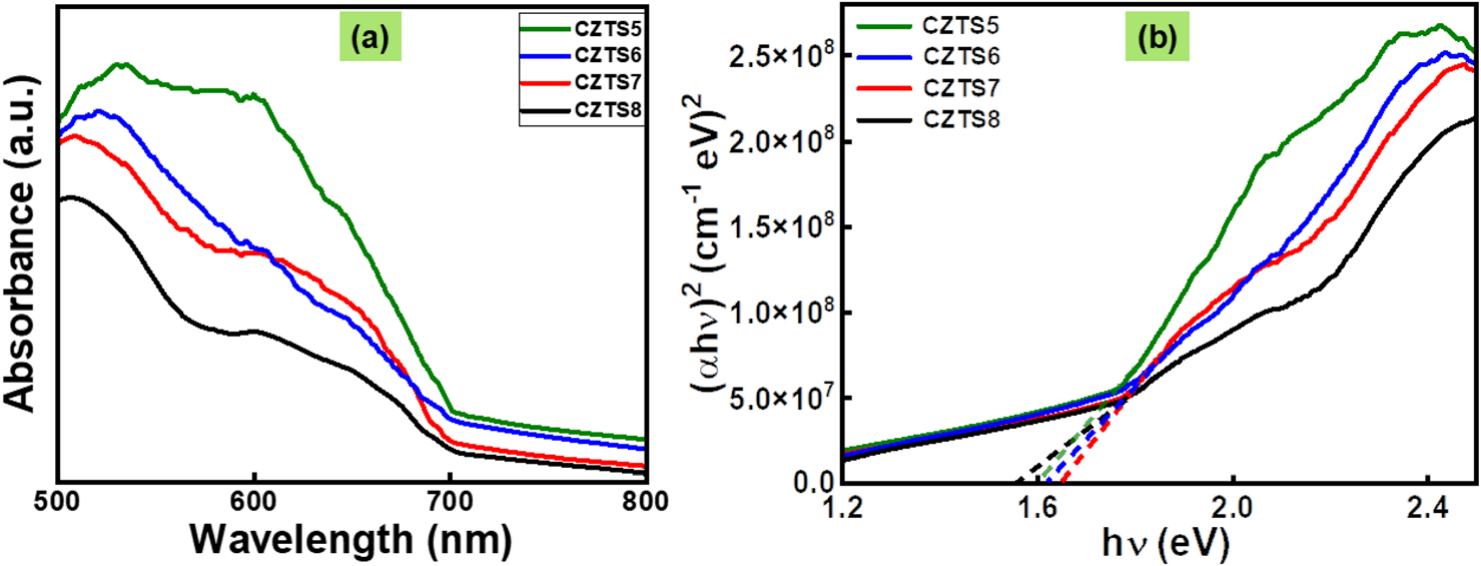
(a) Absorption spectra of CZTS5, CZTS6, CZTS7 and CZTS8 samples and (b) Tauc plot used to estimate the band gap of CZTS5, CZTS6, CZTS7 and CZTS8 samples.

To explore the interaction between light and matter, UV-Visible spectroscopic measurements were conducted at ambient temperature. All CZTS samples with varying sulphur concentrations exhibit absorption in the visible region, as indicated by the black hue of the samples and the absorption edges extending into the visible spectrum. Comparing the absorption curves, it can be observed that absorbance is highest for CZTS5 and lowest for CZTS8 (Fig. 4(a)). The change in the absorbance with sulfur concentration could be correlated with the structural and morphological changes of the CZTS nanomaterials, given the improvement or decline of crystallinity.^31^ It is well demonstrated that a change in absorbance is due to the shape effect and defects in the structure.^39^

By analysing the optical absorbance data, one can establish a correlation between the optical absorption coefficient *α* and photon energy *hυ*, which can be represented graphically *via* the Tauc equation:

3 *αhυ* = *A* (*hυ* − *E*_g_)^*n*^

In this scenario, *A* remains constant, *υ* represents the light frequency employed, and *n* denotes the characteristics of the band transition. If *n* equals 1/2, then a direct allowed transition is indicated. If it equals 3/2, then a direct forbidden transition is suggested. In the case of an indirect permissible transition, a value of 2 is applied, whereas for an indirect forbidden transition, a value of 3 is utilised. This transition is of the direct allowed type, as the values of *n* are 1/2. Finding the band gap, *E*_g_, is as straightforward as sketching the straight line from the graph of (*αh*υ)^2^*vs. h*υ at *α* = 0.

Fig. 4(b) depicts the evolution of the band gap for the samples CZTS5, CZTS6, CZTS7, and CZTS8, which is further encapsulated in Table S2 (SI). The data indicates a clear trend where the optical band gap values rise from 1.60 eV to 1.66 eV, followed by a decline to 1.56 eV as the sulfur content in the samples is increased from a molar ratio of 5 to 8. The decreasing trend followed by the band gap arises due to a decrease in crystallite size and an increase in microstrain, which leads to a decline in crystallinity, thus a red shift occurs for CZTS5 to CZTS7 samples. Now, for the CZTS8, which is rich in sulfur, breaks the trend, and its bandgap decreases to 1.56 eV. This decrease in bandgap may be due to the presence of defective states, which may arise due to the presence of secondary phases, as is evident from Raman analysis.^39^

### 3.5 Morphological and energy dispersive X-ray (EDX) analysis

**Fig. 5:**
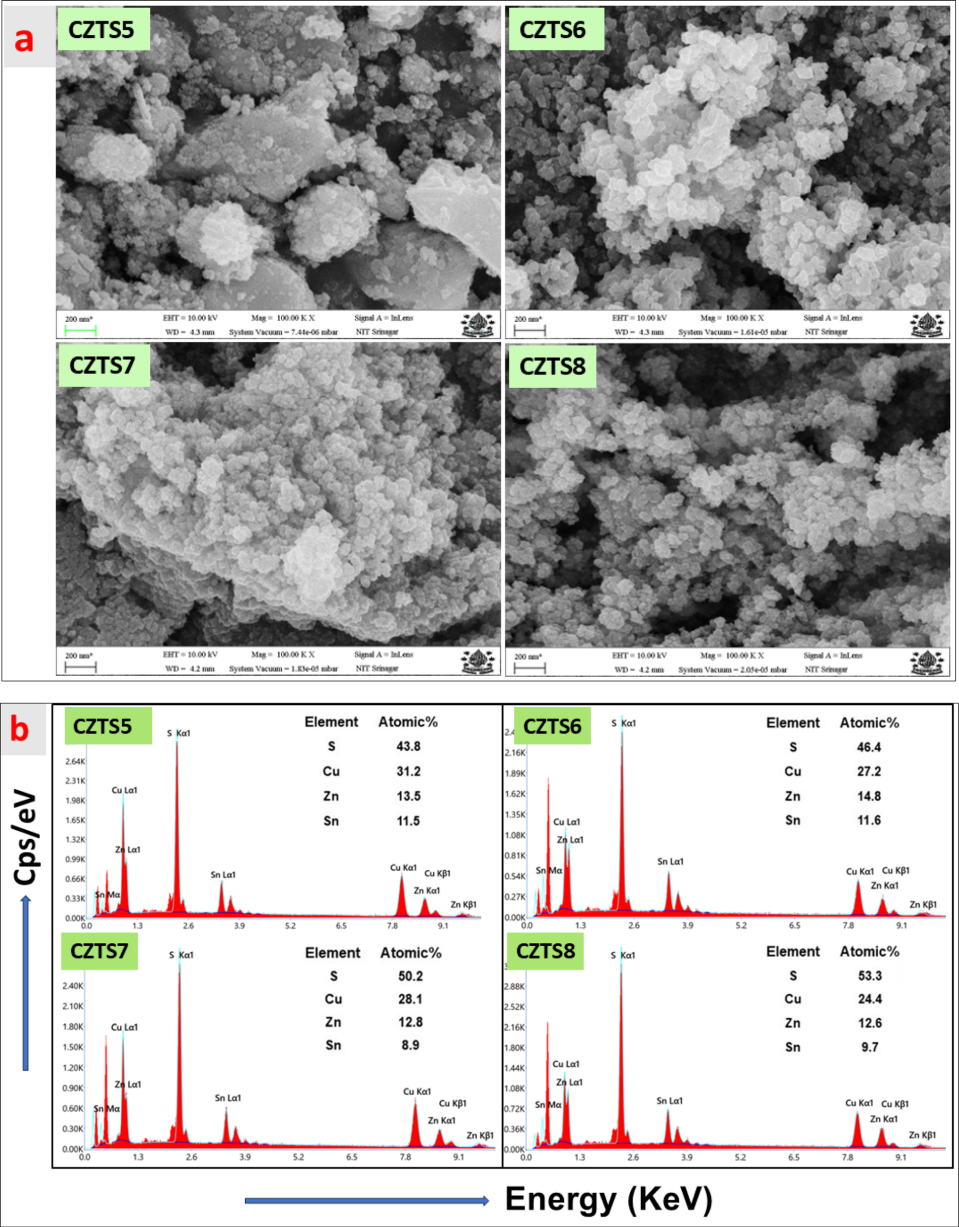
(a) FESEM image of CZTS5, CZTS6, CZTS7 and CZTS8 nanomaterials. (b) Illustrates the energy dispersive spectroscopy (EDS) mapping of CZTS5, CZTS6, CZTS7, and CZTS8 samples.

**Fig. 6:**
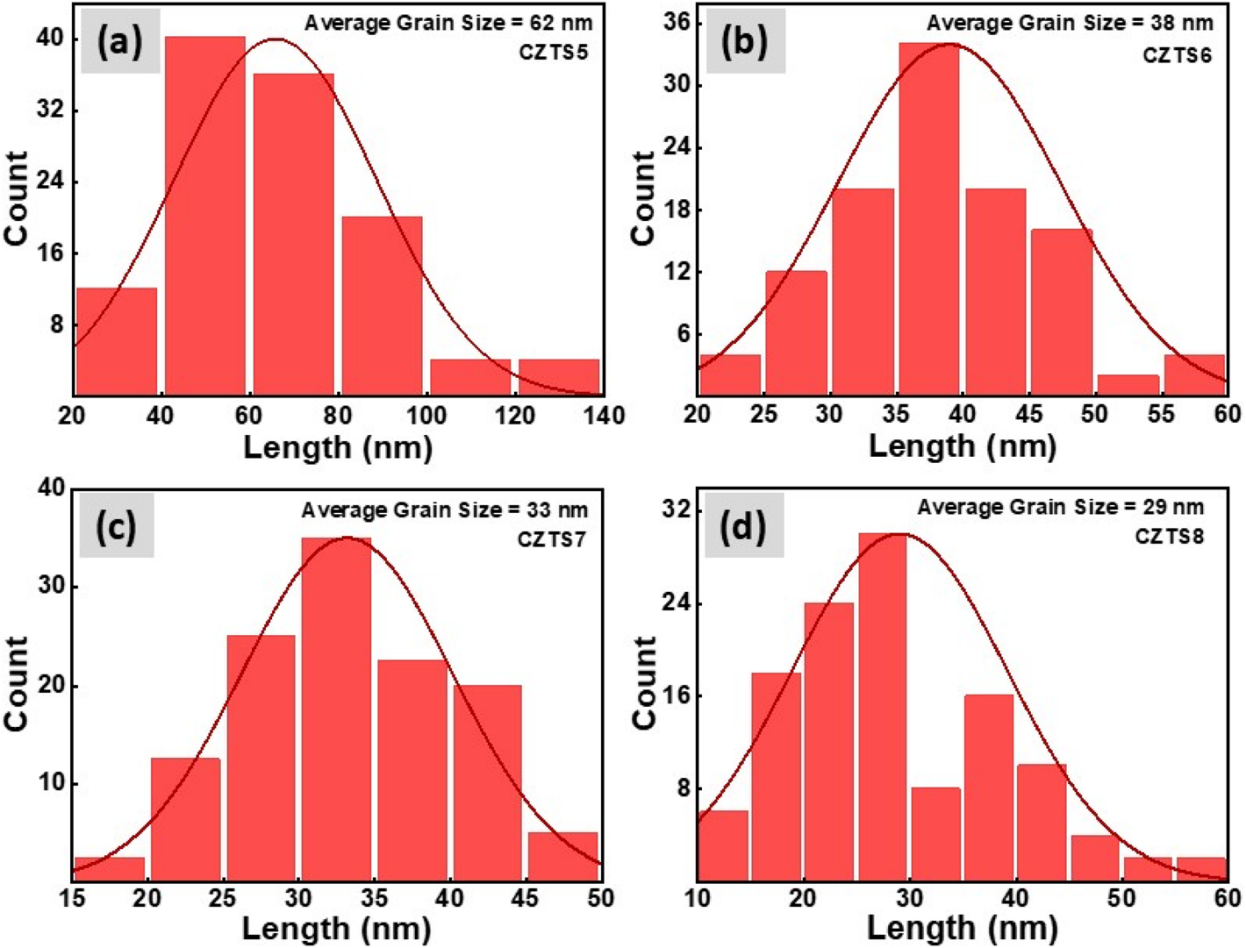
Grain size histogram of (a) CZTS5, (b) CZTS6, (c) CZTS7, (d) CZTS8.

FESEM measurements were conducted to analyse the alterations in the morphology of the samples resulting from variations in sulphur content. The elevated sulphur concentration plays a crucial role in influencing the morphology of CZTS nanomaterials, leading to enhanced or declined structural stabilisation. Fig. 5(a) illustrates the top view of FESEM images for the synthesised CZTS5, CZTS6, CZTS7, and CZTS8 samples. The CZTS5 sample, as illustrated in Fig. 5(a) (CZTS5), exhibits the formation of larger-sized grains, characterised by significant agglomeration and an irregular growth pattern. This behaviour could be linked to an inadequate quantity of sulphur as documented by An *et al.*^40^ As the quantity of sulphur was augmented in samples CZTS6, CZTS7, and CZTS8 illustrated in Fig. 5(a), the surface exhibited increased porosity, a more uniform grain distribution, a reduction in agglomeration, and a decrease in grain size. The observed regularity can be associated with improvements in the morphological properties of CZTS nanomaterials, potentially resulting from optimal stoichiometry or the presence of a Cu_2_S secondary phase.^29^ The observed trend of diminishing grain size aligns with the decrease in crystallite dimensions as the concentration of sulphur escalates. The overall average grain size for all samples was determined using ImageJ software. The histogram in Fig. 6 shows that the average grain size decreases from 62 nm to 29 nm while increasing the sulfur concentration. The decrease in grain size, increase in porous nature, and regularity of morphology will enhance the supercapacitor property.^41^

Fig. 5(b) presents the energy dispersive spectroscopy (EDS) mapping for all the examined samples. The spectrum confirms the compositional integrity of the synthesised nanomaterials, showing no peaks that indicate the presence of additional impurities. The inset of Fig. 5(b) illustrates the distribution of the current components across all samples. The atomic percentage of sulphur rises in direct relation to the increase of sulphur in the reaction medium.

### 3.6 X-ray photoelectron spectroscopy (XPS)

**Fig. 7:**
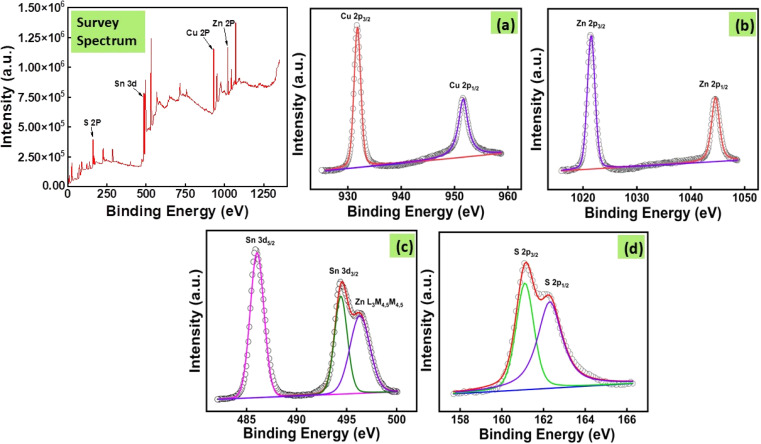
XPS plots of the elements present in the CZTS8 sample with (a) Cu, (b) Zn, (c) Sn, and (d) S.

The above figure presents the XPS plots of all the elements present in CZTS8 nanomaterial. To achieve accurate peak calibration, a carbon reference (C 1s, binding energy 284.6 eV) was employed. Fig. 7(a) displays two significant peaks in the Cu 2p core level spectra, with binding energies of 931.8 eV and 951.6 eV, which are associated with Cu 2p_3/2_ and 2p_1/2_, respectively.^42^ Consistent with the typical split of 19.9 eV, the peak split obtained is 19.8 eV, which indicates the presence of Cu(i).^43^ Moreover, there was an absence of any satellite peak detected at higher binding energy within the Cu 2p spectra. Fig. 7(b) presents the high-resolution spectrum of Zn 2p, showcasing peaks at binding energies of 1021.4 eV and 1044.6 eV, which correspond to Zn 2p_3/2_ and Zn 2p_1/2_, respectively. The measured peak separation is 23.2 eV.^44^ The observed separation aligns precisely with the standard peak split of 23.2 eV, indicating the presence of Zn(ii).^42,43^Fig. 7(c) displays three unique peaks positioned at binding energies of 486.0 eV, 494.5 eV, and 496.6 eV. The initial peak is attributed to Sn 3d_3/2_, while the 494.5 eV peak is associated with Sn 3d_1/2_. The separation between the distinct peaks of Sn 3d_3/2_ and Sn 3d_1/2_ is measured at 8.5 eV, aligning closely with the standard peak splitting, which suggests the presence of Sn(iv).^44^ The final peak observed at 496.6 eV corresponds to the Zn L_3_M_4_,_5_M_4,5_ Auger peak.^43,44^ The core level spectrum of sulphur shown in Fig. 7(d) displays two significant peaks, S 2p_3/2_ and S 2p_1/2_, at binding energies of 161.1 eV and 162.3 eV, respectively. The measured separation of 1.2 eV between these peaks corresponds with the expected range of 160–164 eV for sulphur in sulphide phases, indicating that sulphur is present in the S^2−^ state.^42^

The atomic percentage of each element in the CZTS8 nanomaterial was determined from the corresponding peak area in the XPS spectrum using the equation provided below.4
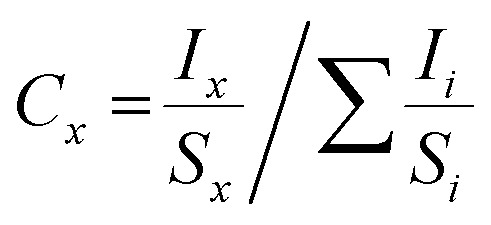


In this framework, *C* denotes atomic concentration, *x* signifies the species of interest, *I* correspond to the peak area, *S* represents the sensitivity factor, and *i* encompasses all potential species. The observed ratio of Cu : Zn : Sn : S approximates 2 : 1 : 1 : 4 with notable precision. Table S2 illustrates the atomic percentage derived from the XPS spectrum for all elements in the CZTS sample. The ratio of copper to the sum of zinc and tin is 0.957, while the ratio of sulphur to the total of copper, zinc, and tin is 1.07, which aligns closely with the results obtained from the EDS spectra.

### 3.7 Brunauer–Emmett–Teller (BET) analysis

**Fig. 8:**
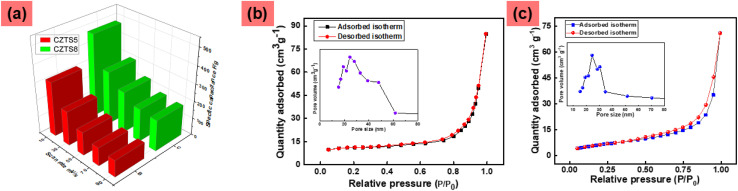
(a) Presents a specific capacitance *versus* scan rate plot for CZTS5 and CZTS8 electrodes, the nitrogen adsorption–desorption isotherms, and (inset) the pore size distribution of CZTS5 (b), and CZTS8 (c).

Based on the FESEM images, CZTS8 exhibits the smallest grain size and pore characteristics compared to all the other samples, including CZTS5. The Brunauer–Emmett–Teller Analysis will facilitate an accurate assessment of the surface area and pore volume of the synthesised CZTS8 and CZTS5 nanomaterials. When it comes to supercapacitors, the sample's porous properties and effective surface area are paramount. As shown in Fig. 8(b) and (c), the nitrogen adsorption–desorption studies were carried out in relation to relative pressure. The graph shows that both samples' sorption curves include a hysteresis loop, which is consistent with a mesoporous surface. The sample's specific surface area, determined *via* the BET method, which was found to be 46.57 m^2^ g^−1^ and 61.48 m^2^ g^−1^ for CZTS5 and CZTS8, respectively.^30,45^ The pore size distribution, depicted in Fig. 8(b) and (c) (inset), was measured to be 17.5 nm and 21.6 nm for CZTS5 and CZTS8, respectively, using the Barrett–Joyner–Halenda (BJH) method. The augmented surface area and porous architecture of the material promote a significant interface between the electrode and electrolyte, thereby improving the diffusion of electrolyte ions, which is essential for redox reactions. The mesoporous structure possesses the capability to significantly improve the electrochemical performance of supercapacitors.

## 4 Electrochemical analysis

**Fig. 9:**
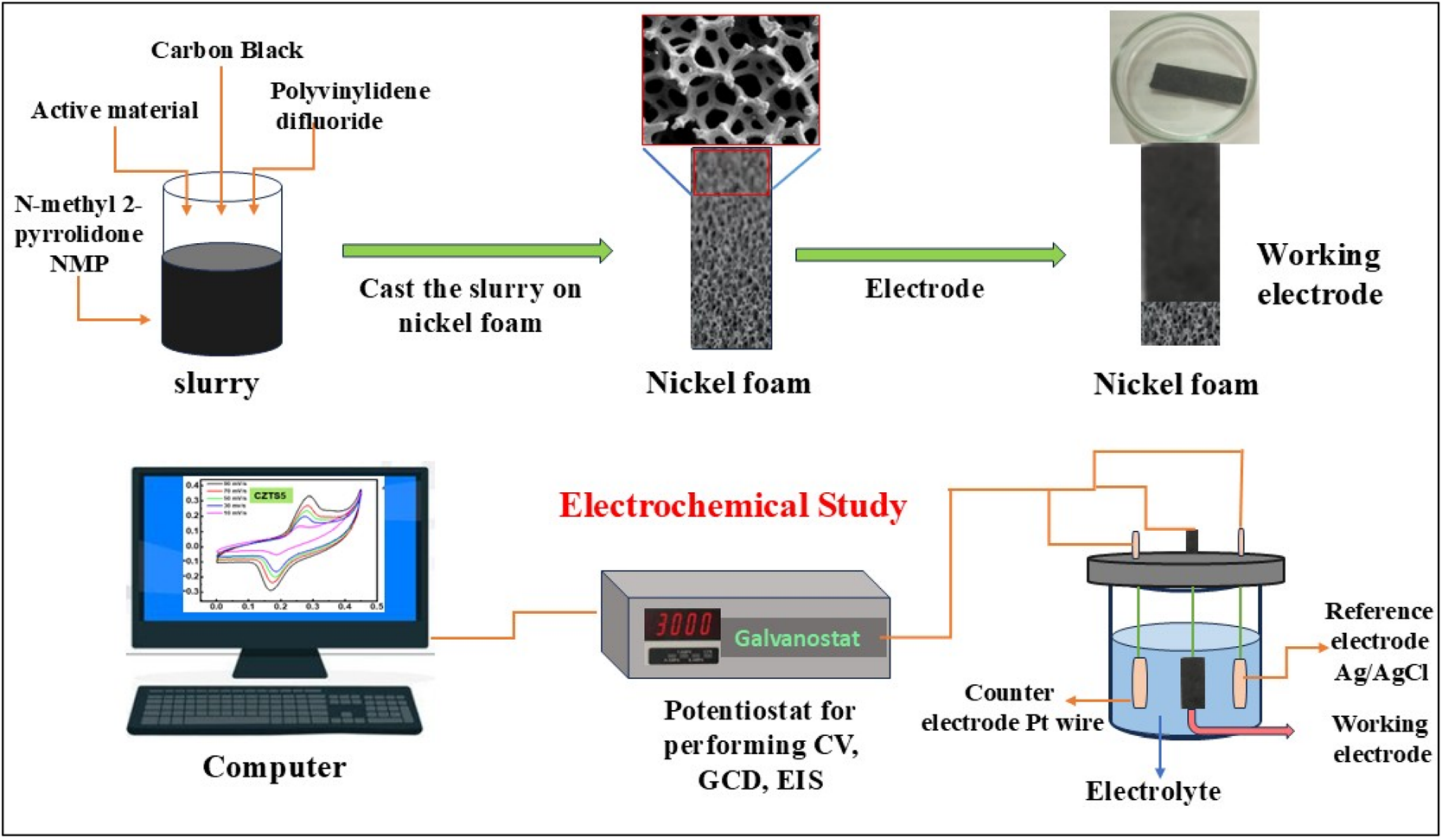
Schematic of the fabrication of the electrode and device operation of the electrochemical work station.

The capacitive properties of the fabricated electrode were investigated through an electrochemical system comprising three key components: an electrochemical cell, an electrochemical work station, and a computer. Electrochemical cell (Fig. 9) is configured with a three-electrode system, working electrode (WE), reference electrode (RE), and counter electrode (CE). In the above Fig. 9, CE and RE are set separately to maintain a stable reference potential, independent of the system's current flow. The potential is measured between the WE and RE, while the current flows between the WE and CE, enabling the precise evaluation of the working electrodes' electrochemical properties. The pseudocapacitive behaviour of CZTS arises from reversible faradaic redox reactions involving its transition metal cations, mainly Cu^+^/Cu^2+^ and, to a lesser degree, Sn^2+^/Sn^4+^, taking place at or near the electrode–electrolyte interface in alkaline media (KOH). During the charging process, Cu^+^ ions are oxidised to Cu^2+^, resulting in the release of electrons. Simultaneously, OH^−^ ions from the electrolyte adsorb onto the CZTS surface to maintain charge neutrality. A small contribution comes from the redox transition of Sn^2+^ to Sn^4+^, especially at surfaces that are rich in defects or have an excess of sulphur, further improving charge storage capabilities. The discharge phase sees a reversal of these redox processes, facilitating cyclic and reversible energy storage.

**Fig. 10:**
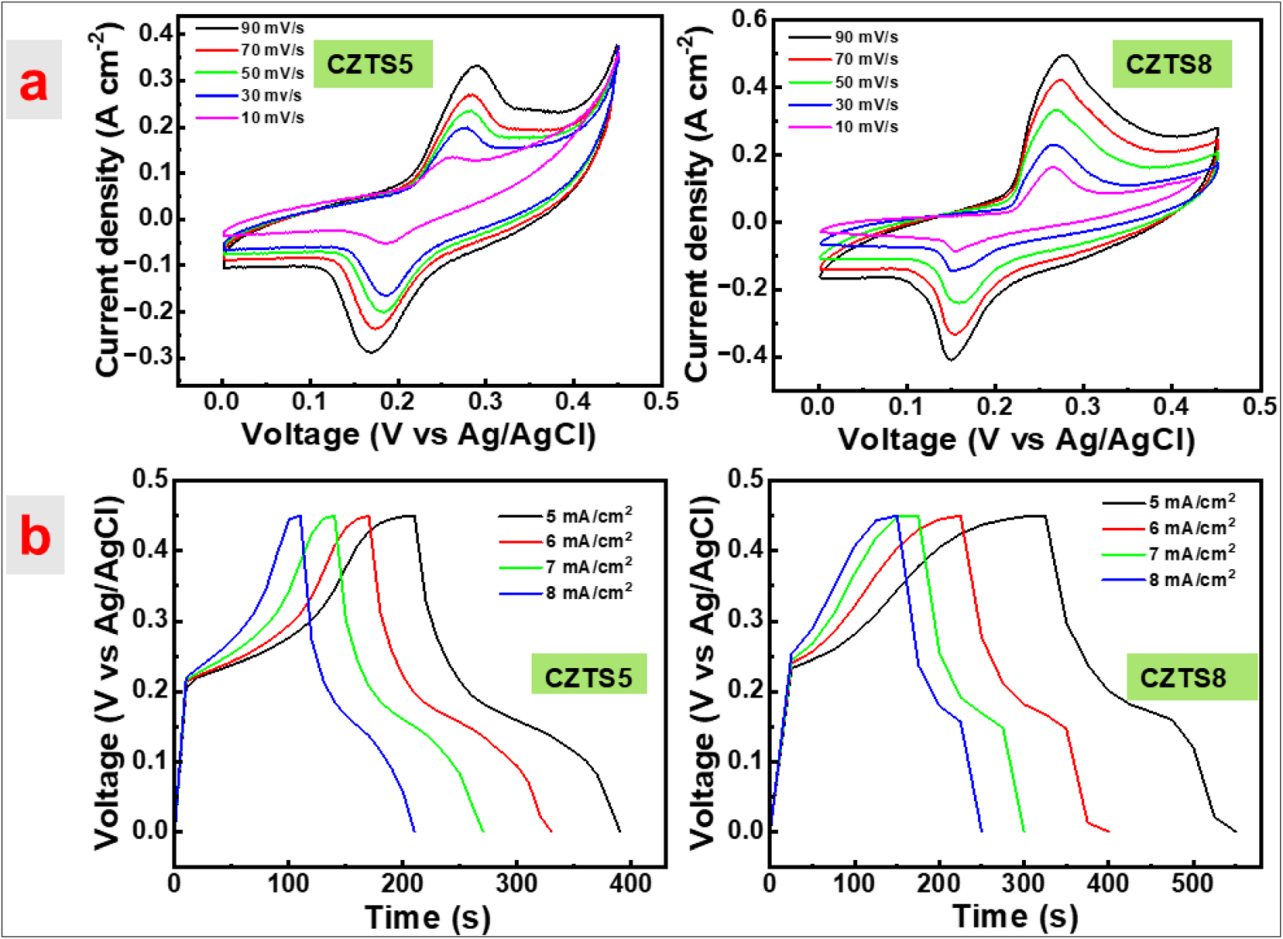
(a)CV curves of CZTS5 and CZTS8. (b) Charge–discharge curve at various current densities CZTS5 and CZTS8.

The electrochemical performance of the CZTS5 and CZTS8 samples was evaluated through cyclic voltammetry (CV), galvanostatic charge–discharge (GCD), and electrochemical impedance spectroscopy (EIS). In these characterisations, a 1 M KOH solution functioned as the electrolyte. Fig. 10(a) present the cyclic voltammetry curves for the CZTS5 and CZTS8 samples, respectively, across varying scan rates from 10 mV s^−1^ to 90 mV s^−1^ inside the potential window of 0 to 0.5 V, compared to an Ag/AgCl electrode. The redox responses of CZTS5 and CZTS8 was validated through the identification of two separate redox peaks, demonstrating a shift towards higher positive (oxidation) and lower negative (reduction) potentials as scan rates increased. This phenomenon is associated with electric polarisation, resistive effects related to the electrode, non-reversible reactions, and enhanced ion kinetics as the scan rate increases.^46^ The swift kinetics led to CV profiles demonstrating a uniform voltammogram shape at elevated scan rates, suggesting that the electrode material exhibits pseudo-capacitance behaviours and improved rate performance in both samples.^46^ The specific capacitance (*C*_sc_) of the samples was derived through the calculation of CV curves using the following equation:^21^5
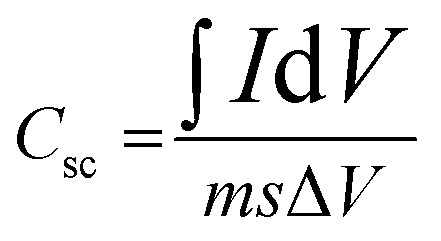


In this scenario, ∫*I*d*V* signifies the area under the CV curve, with *m* indicating the mass of the active material in grams (g), positioned on the working electrode, *s* representing the scan rate (mV s^−1^), and Δ*V* illustrating the potential window (V).

The specific capacitance (*C*_sc_) values determined at scan rates of 10 mV s^−1^, 30 mV s^−1^, 50 mV s^−1^, 70 mV s^−1^, and 90 mV s^−1^ for CZTS8 were 481 F g^−1^, 308 F g^−1^, 239 F g^−1^, 191 F g^−1^, and 181 F g^−1^, while for CZTS5, the values were 316 F g^−1^, 199 F g^−1^, 135 F g^−1^, 106 F g^−1^, and 97 F g^−1^, respectively. The specific capacitance (*C*_sc_) exhibits a decrease at elevated scan rates in both samples, as illustrated in Fig. 10(a). The observed decline can be attributed to the constrained movement of electrolytic ions within the matrix at elevated scan rates, leading to a diminished availability of active sites for the intercalation process.^21,46^ Decreasing scan rates provides ions with a greater temporal window to navigate the tiny pores of the active material, often leading to improved charge storage capacity. At increased scan rates, the constraints imposed by ion diffusion restrict storage potential, resulting in a decrease in capacity.^21^ The CZTS8 exhibits a larger area (elevated *C*_sc_ value) under the CV curve in comparison to CZTS5, a result of enhancements in porosity, surface area, and conductivity that facilitate effective ion transport.^41^ The reduction in grain size of CZTS8 may result in an increased surface-to-volume ratio when compared to CZTS5. This enhancement facilitates a larger electroactive surface area, thereby promoting the efficiency of electrochemical reactions. The CZTS8 sample's electrochemical characteristics may be impacted by the existence of the Cu_2_SnS_3_ (CTS) secondary phase found in the Raman study. Cu_2_SnSn has electrochemically active Cu and Sn species that can take part in redox reactions akin to those in CZTS, and it is known to behave in a semiconducting manner. By adding more redox-active sites, especially through the Cu^+^/Cu^2+^ and Sn^2+^/Sn^4+^ transitions, this phase may improve charge storage capacity and contribute favourably to pseudocapacitance. Analysis using UV-Vis and Tauc plots indicated that an increase in sulphur content results in a decrease in the optical band gap of CZTS8. The reduction in band gap improves electrical conductivity and accelerates charge transfer kinetics, facilitating quicker and more efficient faradaic and non-faradaic processes at the interface between the electrode and electrolyte.^21,47^

The specific capacitance of the fabricated electrodes was assessed *via* galvanostatic charge–discharge (GCD) measurements performed within a potential range of 0–0.5 V, employing current densities from 5 to 8 mA cm^−2^, as depicted in Fig. 10(b). The nonlinear configuration of the GCD profile, characterised by humps, indicates the presence of faradaic effects associated with the constructed electrode across varying current density values. The equation provided below was utilised to compute the *C*_sc_ values.^21^6
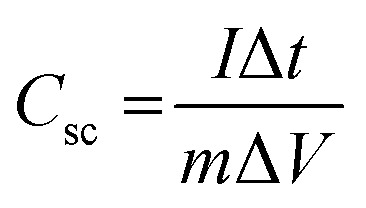
Here, *I* represent current density (A), Δ*t* denotes the charge–discharge time (s), Δ*V* is the potential window (V), and *m* stands for mass of active material (g).

For the CZTS 8 sample, the computed values of *C*_sc_ were 312, 257, 213, and 195 F g^−1^ corresponding to current densities of 5, 6, 7, and 8 mA cm^−2^, respectively. In the case of CZTS5, the calculated *C*_sc_ values were 271, 256, 240, and 205 at current densities of 5, 6, 7, and 8 mA cm^−2^, respectively. The results obtained demonstrate that the decrease in *C*_sc_ values was inversely related to the increase in current densities. This outcome can be attributed to: (i) at elevated velocities, the electron material exhibited an inability to react with precision; (ii) with an increase in current density, the redox reaction at the electrodes became confined to the outer surfaces, suggesting a reduction in capacitance; and (iii) due to the production method of CZTS, an anhydrous surface was formed, which obstructed cation transfer, leading to a swift decline in capacitance.^41,48,49^ The capacitance values of the CZTS8 electrode exceed those of the CZTS5 electrode when measured at 5 mA cm^−2^. This outcome can be ascribed to the favourable crystallite size, grain size, narrow bandgap, and surface area of sulfur-rich CZTS8 in contrast to CZTS5.^41^ The variation in sulphur content exerts an indirect influence on the specific capacitance values derived from GCD analysis. The variations in sulphur lead to alterations in crystallite size, grain size, optical band gap, and also enhance porosity and the surface-to-volume ratio. The greater the exposure of the active material's surface, the higher the concentration of reactive species, leading to an increase in specific capacitance. Moreover, a decrease in band gap enhances electrical conductivity and speeds up charge transfer kinetics, enabling faster and more efficient faradaic and non-faradaic processes at the interface between the electrode and electrolyte.^21^

**Fig. 11:**
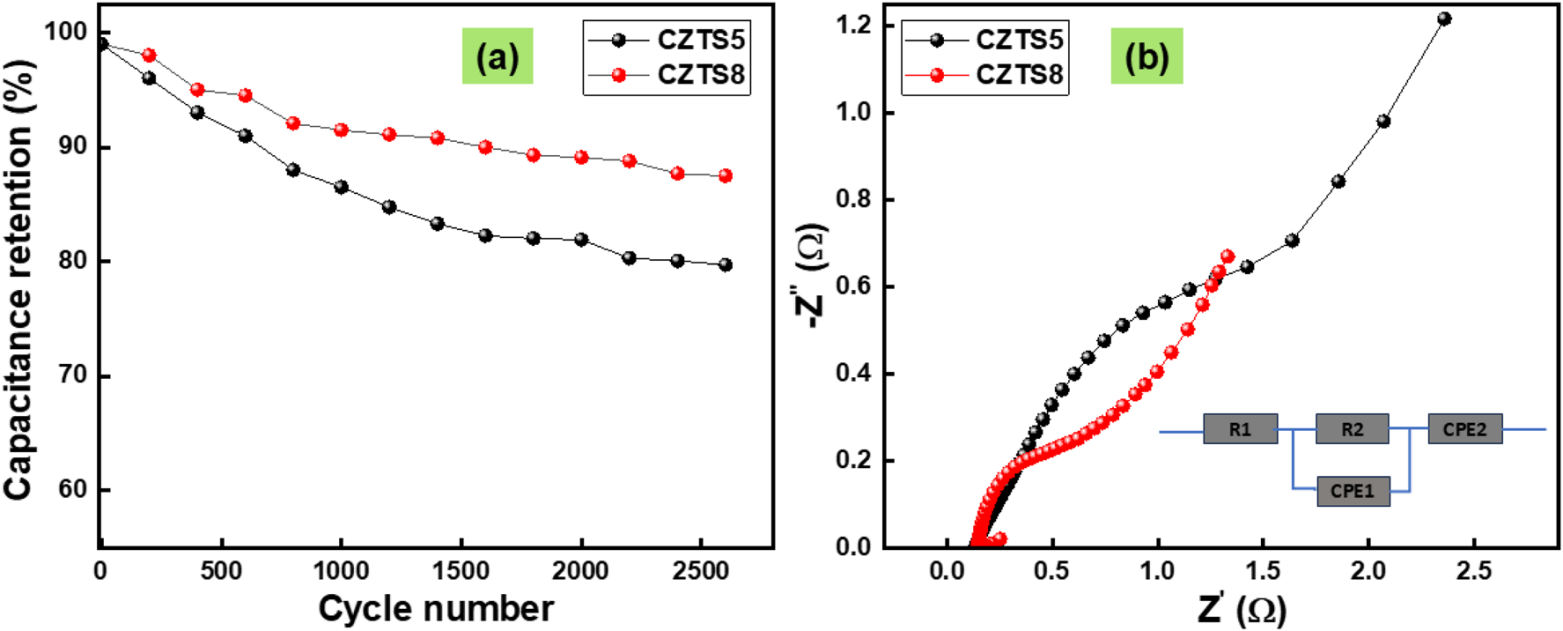
(a) Capacitance retention (%) plotted against cycle number, (b) electrochemical impedance measurements.

Cycle stability is essential for comprehending the demands placed on supercapacitor electrodes in real-world applications. Fig. 11(a) demonstrates the variation in specific capacitance as the cycle number increases for CZTS5 and CZTS8 at scan rates of 30 mV s^−1^, inside the operating voltage window of 0–0.5 V in a 1 M KOH electrolyte. The capacitance in question exhibits a reduction of 11% for CZTS8 and 18% for CZTS5 following 2600 cycles. The CZTS electrodes showed good mechanical integrity and stability during cyclic electrochemical testing, despite the lack of a separate mechanical peel or adhesion test. There were no visible indications of delamination or material separation from the substrate before, but slight delamination following repeated cycling, suggesting strong adherence between the CZTS composite layer and the current collector. Carbon black and a polymeric binder were used to guarantee consistent electrode formation and lessen mechanical stress during repeated charge–discharge operations. Further evidence that the electrode structure remained robust and supported good mechanical cohesiveness of the active layer throughout cycling comes from the retention of specific capacitance over several cycles. The quaternary chalcogenide Cu_2_ZnSnS_4_ presents significant potential as a material, paving the way for advancements in the performance of supercapacitor applications. The results demonstrate that CZTS8 shows enhanced retention relative to CZTS5, which can be ascribed to its porous architecture and a greater number of active sites.

The electrochemical behaviour of CZTS5 and CZTS8 electrode samples at the electrode–electrolyte interface was analysed using a Nyquist plot. The electrochemical impedance spectroscopy measurements were conducted across a frequency spectrum of 0.01 Hz to 100 kHz, with the resulting plots illustrated in Fig. 11(a). The graphical representation distinctly illustrates that both EIS plots demonstrate a semicircular arc in the high-frequency domain and a linear progression in the low-frequency domain. The solution resistance (*R*_s_) of the electrochemical system can be ascertained from the intercept observed on the real axis of the plot. The existence of a semicircular region signifies the presence of charge-transfer resistance (*R*_ct_). The plots in Fig. 11, indicate that CZTS8 exhibits a smaller diameter compared to CZTS5, implying reduced charge transfer resistance and consequently enhanced conductivity.^50^ The *R*_s_ and *R*_ct_ resistance characteristics for CZTS5 were 0.14 Ω and 1.53 Ω, whereas the values for CZTS8 were 0.142 Ω and 0.55 Ω. In addition, the extension of the straight line indicated that both materials exhibited capacitive behaviour.

The XRD and FTIR characterisations were done on both the pure material and its electrode after performing electrochemical measurements Fig. S2, (SI). From the XRD pattern spectra display clear peaks corresponding to the crystallographic planes (112), (200), (220), (312), (008), and (332) at diffraction angles of 28.56°, 32.93°, 47.45°, 56.31°, 69.30°, and 76.47°, respectively in pure CZTS. CZTS electrode with nickel foam as substrate shows intense peaks of Ni at 2theta, 44.34 (1 1 1), 51.54 (2 0 0), and 76.09 (2 2 0),^51^ and less intense peaks of CZTS material, Fig. S2. From the FTIR graph, both spectra display distinct absorption bands in the range of ∼500–700 cm^−1^, commonly associated with the vibrational modes of metal–sulfur bonds (Cu–S, Zn–S, and Sn–S), thereby affirming the formation of the CZTS phase.^52^ Subtle variations or intensity discrepancies could likewise be linked to the interactions occurring between CZTS and the substrate or binder employed in the preparation of the electrode. These findings reinforce the structural stability of CZTS after deposition and indicate favourable chemical compatibility with the electrode matrix.

## 5 Conclusion

The solvothermal approach was utilised to successfully execute the synthesis of nanoscale Cu_2_ZnSnS_4_. An investigation was conducted to determine how the concentrations of sulphur affected the structural, optical, morphological, and compositional aspects of the material. The influence of increasing sulphur concentration was initially investigated by means of XRD patterns. The results of this investigation revealed a decrease in crystallite size as the concentration of sulphur increased. Furthermore, the preferential alignment along the (200) and (100) lattice planes provided evidence that the kesterite phase was present. The influence of sulphur on the crystallinity of synthesized CZTS nanomaterials was studied through the parameters such as crystallite size and microstrain. Both electron diffraction and X-ray diffraction (EDS) and X-ray diffraction (XPS) offer support for the compositional ratio of the CZTS nanomaterial, which is calculated using both of these methods. An increase in the surface-to-volume ratio of the nanomaterials was observed in conjunction with an increase in the amount of sulphur, and the nanomaterials exhibited a spherical that was evenly distributed. The optical band gap exhibits a gradual increase from 1.60 eV to 1.66 eV, followed by a decrease to 1.56 eV, as the sulphur content in the samples is varied from a molar ratio of 5 to a molar ratio of 8. The variation in sulfur concentrations affects the structural, optical, and morphological properties, which in turn affect the electrochemical properties. The variation in specific capacitance as a function of cycle number for CZTS5 and CZTS8 was observed at scan rates of 30 mV s^−1^ across a voltage window of 0–0.5 V in a 1 M KOH electrolyte, with data collected over multiple cycles. The specific capacitance decreases by 11% for CZTS8 and 18% for CZTS5 after 2600 cycles. The quaternary chalcogenide Cu_2_ZnSnS_4_ presents significant potential as a material, paving the way for advancements in the performance of supercapacitor applications.

## Author contributions

Roomul Mushtaq: conceptualisation, data curation, formal analysis, investigation, methodology, software, validation, visualisation, writing – original draft, writing – review & editing. Mohd Zubair Ansari: review & editing, resources, supervision.

## Conflicts of interest

The work described in this article could not have been influenced by the author's known conflicting financial interests or personal ties.

## Supplementary Material

RA-015-D5RA04633E-s001

## Data Availability

This article contains all of the data created or examined during this investigation. The supplementary information includes experimental details presented within a schematic figure labelled as Fig. S1, Table S1. Crystallite size (determined through the Scherrer and Williamson-Hall equation), strain, and band gap values of CZTS samples with varying sulphur amounts, Table S2. Atomic %, peak area of the peaks of S 2p, Sn 3d, Cu 2p, and Zn 2p present in CZTS8 sample, and Fig. S2. XRD (a), and FTIR (b) of pure CZTS and CZTS electrode. See DOI: https://doi.org/10.1039/d5ra04633e.
