# Supplementary material for: Investigation of the impact of sulfur on the properties of CZTS nanomaterials for enhanced supercapacitor performance
Source: RSC Adv. 2025 Sep 2;15(38):31447–60. doi: 10.1039/d5ra04633e (PMC12402769; doi:10.1039/d5ra04633e)
Supplement: RA-015-D5RA04633E-s001 [file RA-015-D5RA04633E-s001.pdf]

Supplementary Information (SI) for RSC Advances.
This journal is © The Royal Society of Chemistry 2025

# Investigation of the Impact of Sulfur on the Properties of CZTS Nanomaterials for Enhanced Supercapacitor Performance

**Roomul Mushtaq and Mohd Zubair Ansari**

Department of Physics, National Institute of Technology Srinagar, Hazratbal Srinagar, J&K, 190006 India
Corresponding author email: mhd.zubair1@gmail.com

## Supporting Information

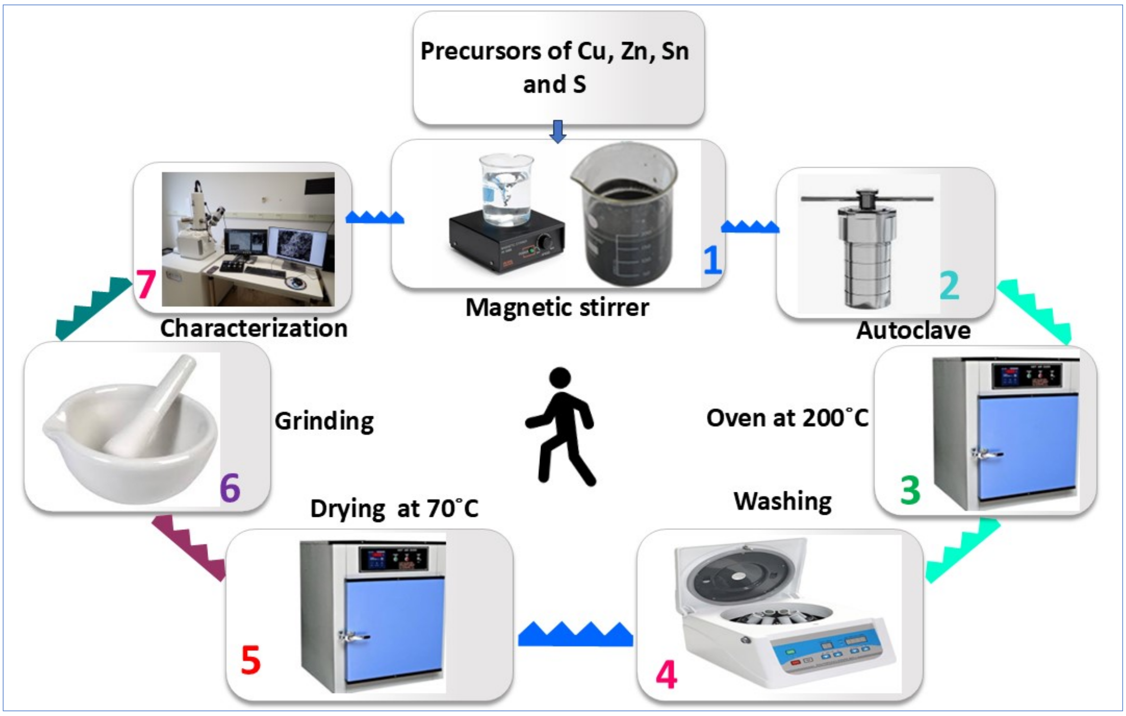


**Figure s1**. The steps for the synthesis of CZTS nanomaterials are presented.

| Sample name | Crystallite size using Scherrer equation | Strain × $10^{-3}$ | Crystallite size using Williamson-Hall equation | Band gap |
|---|---|---|---|---|
| **CZTS5** | 22.35 nm | 2.82 | 28.26 nm | 1.62 eV |
| **CZTS6** | 15.75 nm | 3.50 | 20.38 nm | 1.66 eV |
| **CZTS7** | 14.5 nm | 7.19 | 18.23 nm | 1.69 eV |
| **CZTS8** | 11.3 nm | 9.41 | 15.61 nm | 1.59 eV |

**Table S1**. Crystallite size (determined through the Scherrer and Williamson-Hall equation), strain, and band gap values of CZTS samples with varying sulphur amounts.

| Name | Peak BE | Height CPS | FWHM eV | Area (I) CPS.eV | Atomic % |
|---|---|---|---|---|---|
| S 2p | 161.31 | 205290.46 | 2.96 | 843645.31 | 53.36 |
| Sn 3d | 485.69 | 503032.1 | 2.52 | 1346131.09 | 9.05 |
| Cu 2p | 931.32 | 466567.96 | 2.62 | 2654798.94 | 22.70 |
| Zn 2p | 1021.13 | 339791.33 | 2.73 | 1828593.71 | 14.80 |

**Table S2**. Atomic %, peak area of the peaks of S 2p, Sn 3d, Cu 2p, and Zn 2p present in CZTS190 sample

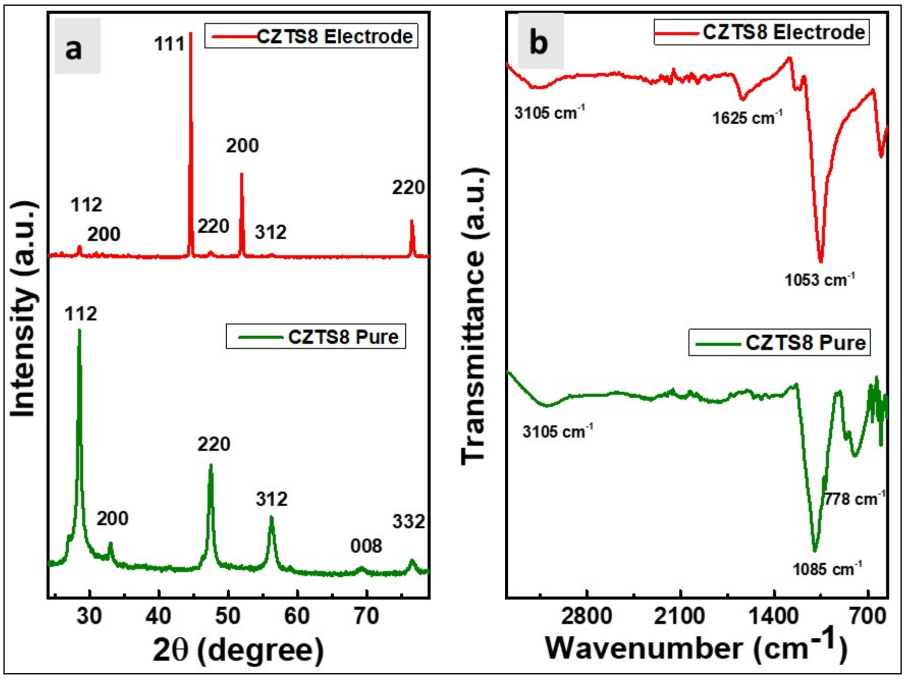


**Figure s2**. XRD (a), and FTIR (b) of pure CZTS and CZTS electrode.
